# High-Performance Porous Organic Polymers for Environmental Remediation of Toxic Gases

**DOI:** 10.1021/acs.langmuir.3c03980

**Published:** 2024-04-04

**Authors:** Mohammad G. Rabbani, Riley K. Sasse, Swayamprabha Behera, Puru Jena, Jian Liu, Praveen K. Thallapally, Timur Islamoglu, Mohammad K. Shehab, Mahmoud M. Kaid, Omar K. Farha, Hani M. El-Kaderi

**Affiliations:** † Department of Chemistry, University of Wisconsin-Platteville, Platteville, Wisconsin 53818, United States; ‡ Department of Chemistry, Pennsylvania State University, University Park, Pennsylvania 16802, United States; § Department of Physics, Kennesaw State University, Marietta Campus, 1100 South Marietta Pkwy, Marietta, Georgia 30060, United States; ∥ Department of Physics, Virginia Commonwealth University, Richmond, Virginia 23284, United States; ⊥ Pacific Northwest National Laboratory, Richland, Washington 99352, United States; ∇ Department of Chemistry, Northwestern University, 2145 Sheridan Road, Evanston, Illinois 60208, United States; ○ Department of Chemistry, Virginia Commonwealth University, Richmond, Virginia 23284, United States

## Abstract

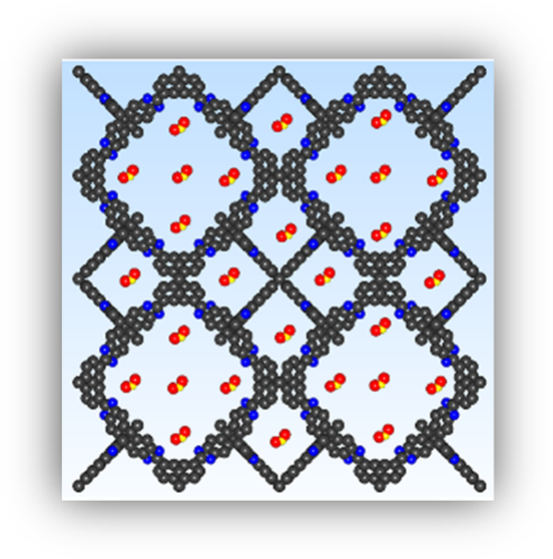

Sulfur dioxide (SO_2_) is a harmful acidic gas generated from power plants and fossil fuel combustion and represents a significant health risk and threat to the environment. Benzimidazole-linked polymers (BILPs) have emerged as a promising class of porous solid adsorbents for toxic gases because of their chemical and thermal stability as well as the chemical nature of the imidazole moiety. The performance of BILPs in SO_2_ capture was examined by synergistic experimental and theoretical studies. BILPs exhibit a significantly high SO_2_ uptake of up to 8.5 mmol g^–1^ at 298 K and 1.0 bar. The density functional theory (DFT) calculations predict that this high SO_2_ uptake is due to the dipole–dipole interactions between SO_2_ and the functionalized polymer frames through O_2_S(δ^+^)···N(δ^–^)-imine and O=S=O(δ^–^)···H(δ^+^)-aryl and intermolecular attraction between SO_2_ molecules (O=S=O(δ^–^)···S(δ^+^)O_2_). Moderate isosteric heats of adsorption (*Q*_st_ ≈ 38 kJ mol^–1^) obtained from experimental SO_2_ uptake studies are well supported by the DFT calculations (≈40 kJ mol^–1^), which suggests physisorption processes enabling rapid adsorbent regeneration for reuse. Repeated adsorption experiments with almost identical SO_2_ uptake confirm the easy regeneration and robustness of BILPs. Moreover, BILPs possess very high SO_2_ adsorption selectivity at low concentration over carbon dioxide (CO_2_), methane (CH_4_), and nitrogen (N_2_): SO_2_/CO_2_, 19–24; SO_2_/CH_4_, 118–113; SO_2_/N_2_, 600–674. This study highlights the potential of BILPs in the desulfurization of flue gas or other gas mixtures through capturing trace levels of SO_2_.

## Introduction

The emission of acidic gases from fossil fuel consumption is a serious threat to human health and the environment.^1,2^ Apart from carbon dioxide’s (CO_2_) greenhouse effect, which contributes to global warming and climate change, the effects of sulfur dioxide (SO_2_) on the environment are even more hazardous. Particularly, the acid rain caused by SO_2_ is responsible for deforestation and threatens animal life cycles.^3^ According to the National Library of Medicine, the exposure of SO_2_ over 100 ppm in various species is deadly,^4^ and its concentration should not exceed 0.5 ppm more than once per year for 3 h according to the US EPA.^5^ Therefore, it is highly desirable to develop efficient materials that can selectively remove SO_2_ from the source gas mixtures. The existing technologies for SO_2_ capture include limestone scrubbing,^6−9^ ammonia scrubbing,^10^ and absorption by organic solvents and ionic liquids.^6−9,11^ However, sulfate byproducts, acidic wastewater, and solvent volatilization and recycling at high temperatures represent serious concerns that still need to be addressed. The use of porous solid adsorbents in capturing toxic gases is a promising method and has been extensively studied over the last two decades. In recent years, there has been a significant interest in studying the SO_2_ capture by synthesized porous materials both purely organic structures and organic–inorganic hybrid structures commonly known as metal–organic frameworks (MOFs).^12−19^ In particular, MOF-177 that has an ultrahigh specific surface area (4100 m^2^ g^–1^) possessed a remarkable SO_2_ uptake of 25.7 mmol g^–1^ at 293 K and 1.0 bar.^20^ Similarly, NOTT-202a (2220 m^2^ g^–1^) exhibited 13.6 mmol g^–1^ at 268 K.^14^ Although MOFs are promising because of their high surface areas and highly ordered structures, the acidic nature of SO_2_ particularly in the presence of moisture may destabilize the frameworks. In that aspect, porous organic polymers that are linked with strong covalent bonds appear to be more promising in environmental remediation.^21−23^ A recent report on benzimidazole-derived carbons (BIDCs) showed very high SO_2_ sorption capacity up to 21.42 mmol g^–1^ at 298 K and 1 bar.^12^ BIDCs were made by the pyrolysis of imidazole in the presence of KOH at high temperatures. BIDC-3-800, which was made by pyrolysis at 800 °C, is highly porous with a surface area of 3750 m^2^ g^–1^ and exhibited the best performance of SO_2_ uptake (21.42 mmol g^–1^ at 298 K and 1 bar), while BIDC-2-700 prepared at 700 °C (surface area 1200 m^2^ g^–1^) showed SO_2_ uptake of 10.25 mmol g^–1^ at the same condition. High surface area and heteroatom functionality are expected to play important roles in high SO_2_ uptake. Functionalized polymers of intrinsic microporosity (PIMs) also showed high SO_2_ uptake (7.32 mmol g^–1^ at 298 K and 1 bar).^24^ The recent report on SO_2_ capture by cage molecules showed that the tertiary amine has higher SO_2_ capture capabilities than the secondary amine or imine functionalities.^16^ SO_2_ has a very high affinity toward primary amines, and it can be used as the connector between two primary amine functional groups through the formation of sulfonamide linkage.^25^ These findings encouraged us to study the effect of heteroatoms, particularly imidazole rings, on the selective SO_2_ capture. Over the past decade, we have reported a series of benzimidazole-linked polymers (BILPs), which are thermally and chemically robust under acidic and basic conditions, and they exhibit excellent performance in CO_2_ capture.^26−28^ BILPs also work as promising metal-free photocatalysts for CO_2_ reduction.^29^ Structural features such as high surface area, pore functionality with heteroatoms, and physicochemical stability make BILPs ideal for acidic SO_2_ capture and separation.

**Figure 1:**
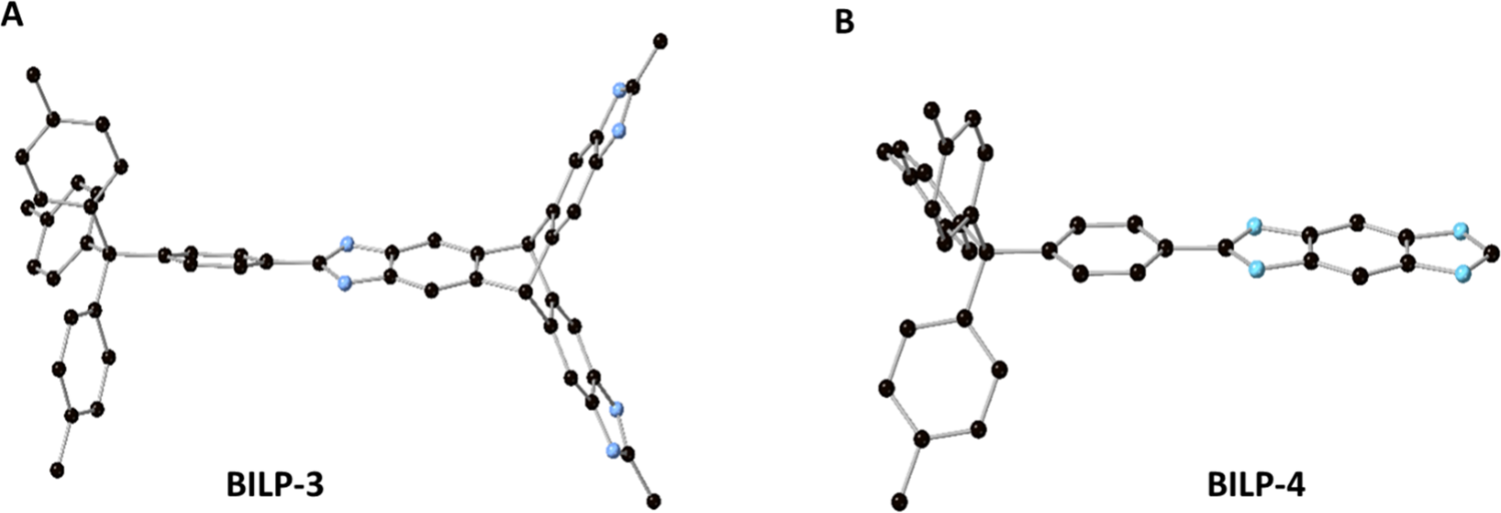
Ball–stick models for the repeating units for BILP-3 (A) and BILP-4 (B) showing the benzimidazole moieties and tetraphenylmethane cores (carbon: black, nitrogen: blue; hydrogen omitted for clarity).

Herein, we studied the effects of the chemical and textural properties of BILPs on SO_2_ capture. Both experimental and theoretical studies have been performed for two representative BILPs, namely, BILP-3 and BILP-4 (Figure 1). We demonstrate that BILPs are well-suited for selective SO_2_ capture because of their high thermal and chemical stability, permanent porosity, and exceptionally high uptake capacity, which are among the highest for all known covalently linked porous organic materials such as COFs, POPs, and cage molecules reported to date. Our experimental and theoretical studies reveal that the electronic nature of the benzimidazole moiety in the polymer frame plays a key role in selective and high SO_2_ uptake.

## Experimental Section

### Materials and Methods

All chemicals, including the monomer 1,2,4,5-benzenetetramine (BTA) used in this research, were purchased from commercial suppliers (Sigma-Aldrich, Acros Organics, or Frontier Scientific) and used as received. Synthesis and characterization of other monomers (tetrakis(4-formylphenyl)methane (TFPM) and 2,3,6,7,14,15-hexaaminotriptycene (HATT)) and polymers BILP-3 and BILP-4 were reported in our earlier publications.^26,27^ Synthesized polymers were characterized by performing thermogravimetric analysis (TGA), Fourier-transform infrared spectroscopy (FT-IR), and X-ray photoelectron spectroscopy (XPS) measurements. The details of these measurements are discussed in Supporting Information. Surface areas and porosity were calculated from nitrogen adsorption isotherms collected using a Quantachrome Autosorb 1-C volumetric analyzer. The SO_2_ uptakes were studied using the facilities at Northwestern University (NU) and Pacific Northwest National Laboratory (PNNL). For volumetric equilibrium SO_2_ uptakes measured at NU, the materials were tested by using pure SO_2_ gas on a Micromeritics 3Flex Surface Analyzer at 298 K equipped with a corrosive resistance-enhanced manifold. The 3Flex was set up in a walk-in fume hood, and the environment was monitored with a SO_2_ sensor. Samples were degassed at 60 °C overnight before any gas adsorption experiments. The recyclability of the material was tested by reanalyzing the material for each toxic gas a second time after activation under vacuum at room temperature to remove adsorbed gas from the previous analysis. The dynamic sorption system used at the PNNL to measure SO_2_ adsorption and desorption isotherms is shown in Figure S1 and is discussed there.

### Computational Methodology

To reduce the computational demand, polymeric BILPs were modeled by terminating the corresponding segment of the benzimidazole-containing units with hydrogen atoms. The segment used for BILP-3 calculation has 3 benzimidazole units, while the segment used for BILP-4 calculation has 2 benzimidazole units (Figure S6). The binding affinities (BE) per SO_2_ of BILP-3 and BILP-4 were calculated using the following equation:
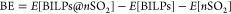
Here, BILPs@*n*SO_2_ represents the BILPs interacting with *n*SO_2_ molecules (*n* is the number of SO_2_ molecules). E[BILPs@*n*SO_2_] is the total energy of BILPs@*n*SO_2_, while E[BILPs] is the energy of the polymer without SO_2_ and E[*n*SO_2_] is the total energy of *n*SO_2_ molecules. The number of SO_2_ molecules (*n*) is chosen based on the number of nitrogen centers within the polymer segment used in the calculation. For BILP-3, *n* values are 3 (half of the total nitrogen centers) and 6 (equal to the total nitrogen centers). Similarly, the number of SO_2_ molecules (*n*) for BILP-4 is 2 (half of the total nitrogen centers) and 4 (equal to the total nitrogen centers). The density functional theory (DFT) calculations were performed using two forms of exchange–correlation potentials: local density approximation functional (LDA)^30^ and hybrid meta exchange–correlation functional, M06.^31^ The M06 functional is particularly important because it accounts for the dispersive forces, which may play a key role in the case of weak interaction between the BILP substrate and SO_2_ molecules. The LDA, on the other hand, overestimates binding^32^ and, in some cases, yields binding energies closer to experiments when interactions are weak. However, this agreement results from the fortunate cancellation of errors as LDA does not include long-range dispersive forces. All calculations were carried out using Gaussian 09^33^ software and 6-311+G*^34^ basis sets.

In obtaining the equilibrium geometries, the SO_2_ molecules were allowed to approach the top and bridge sites of the central ring as well as the top and planar sites of N atoms. The SO_2_ molecule was also aligned horizontally and vertically to the plane of the benzimidazole moiety. Geometry optimization was initially performed with LDA and then further with M06. Minimum in the potential energy surface (PES) was achieved to ensure the most stable geometry. Natural bonding orbital method (NBO)^35^ was used to calculate the atomic charges.

## Results and Discussion

**Scheme 1:**
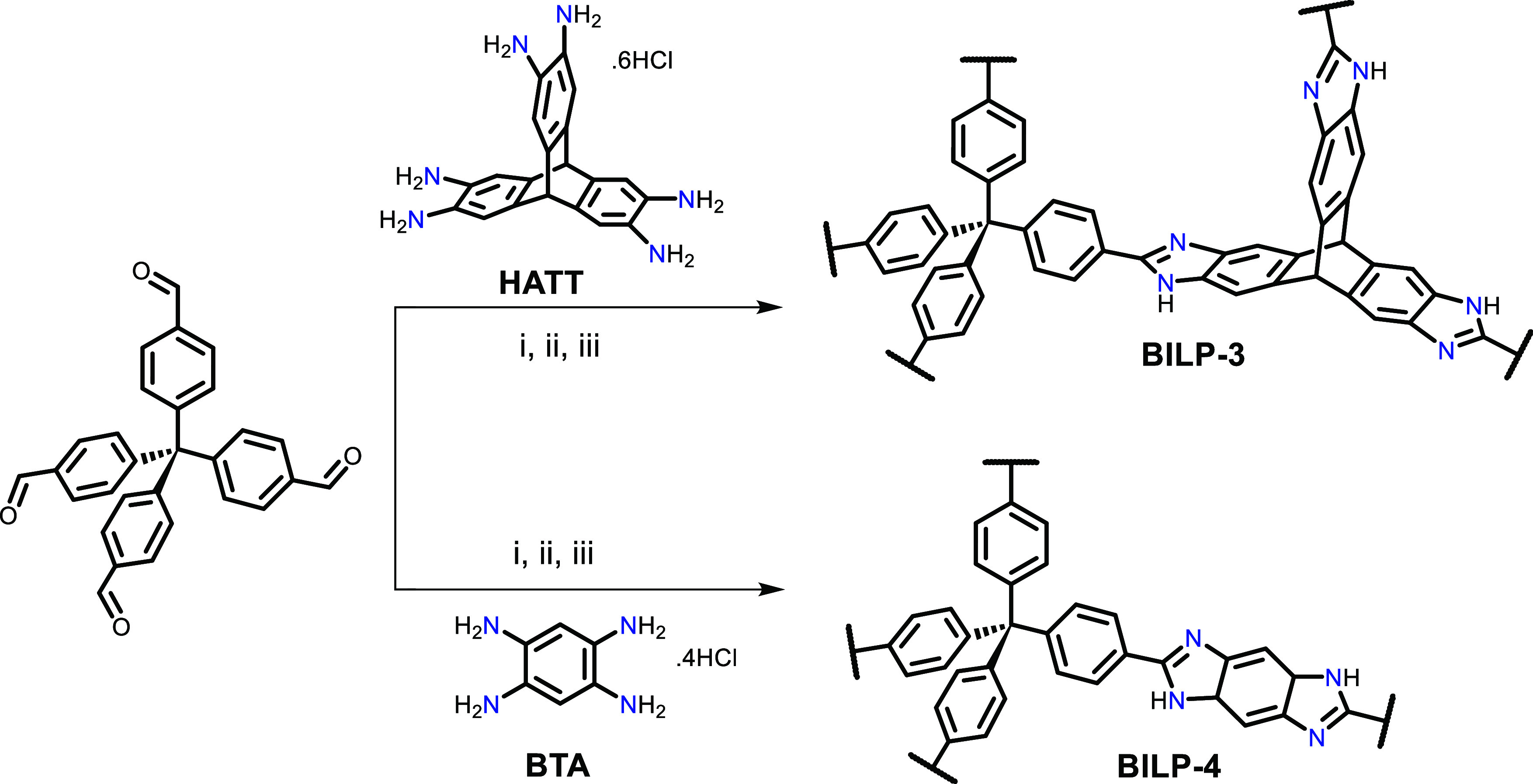
Synthesis of BILP-3 and BILP-4 (i) Anhydrous dimethylformamide (DMF), −30 °C, 3 h, under N_2_ atmosphere; (ii) DMF, room temperature, 6 h, under N_2_ atmosphere; (iii) DMF, 130 °C, 3 days, under O_2_ atmosphere.

The synthesis of BILP-3 and BILP-4 and their porosities were reproduced according to our published methods (Scheme 1).^26,27^ Briefly, condensation reactions between aldehyde and HCl salt form of amine monomers (TFPM and HATT, or BTA) afforded BILP-3 and BILP-4, respectively. The solids were collected as yellow powders in good yields. After washing with aqueous 2 M HCl, 2 M NaOH, deionized water, and acetone, the solid products were dried at first at ambient conditions and then under reduced pressure at 120 °C. Nitrogen sorption isotherms were collected for the activated samples at 77 K. The calculated Brunauer–Emmett–Teller (BET) surface area and pore size distribution using nonlocal-density function theory (NLDFT) agreed well with the reported values: BILP-3 (SA: 1390 m^2^ g^–1^, PSD: 10.6 Å) and BILP-4 (SA: 1220 m^2^ g^–1^, PSD: 10.6 Å). It is worth noting that both BILPs have high chemical and thermal stabilities up to 400 °C based on reported TGA studies.

**Figure 2:**
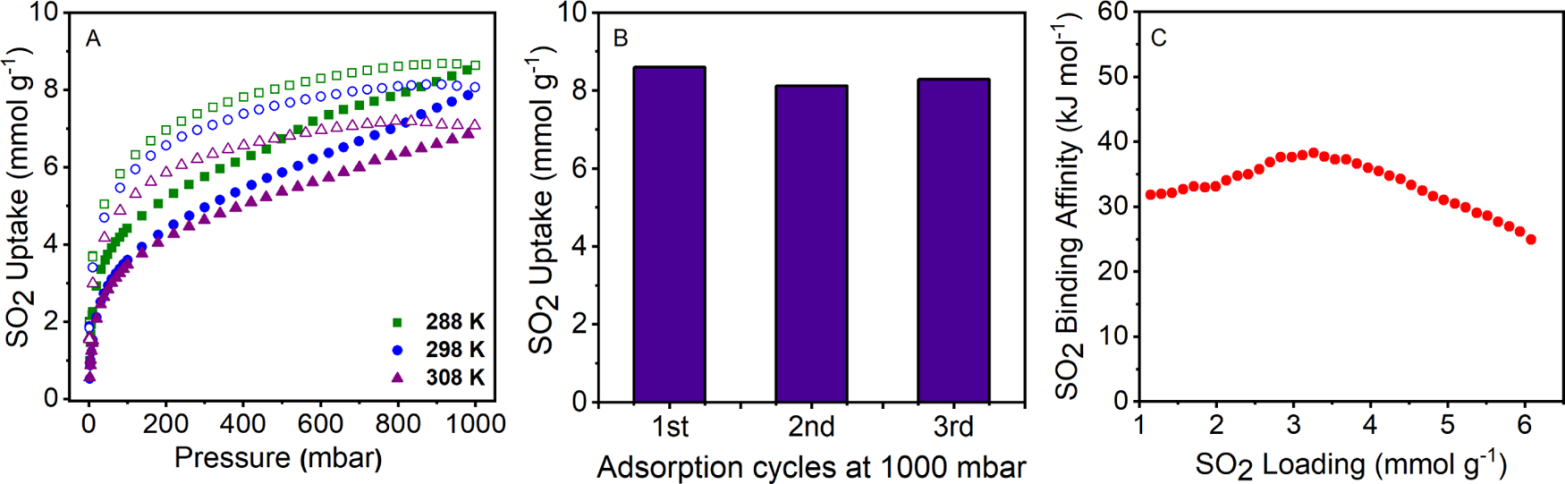
Volumetric SO_2_ adsorption–desorption isotherms for BILP-3 at 288, 298, and 308 K (A), filled (adsorption) and empty (desorption) data. Recyclability of SO_2_ adsorption for BILP-3 at 298 K (B). The binding affinity of SO_2_ in BILP-3 was calculated by using isotherms collected at 288, 298, and 308 K (C).

Volumetric SO_2_ adsorption isotherms for BILP-3 were collected at 288, 298, and 308 K using the facility at Northwestern University and are shown in Figure 2A. Adsorption isotherms were also collected using the dynamic adsorption system for both BILP-3 and BILP-4 at 298 K using the facility at Pacific Northwest National Laboratory, and they are shown in Figure S2A. It has been found that there is a slight deviation in the SO_2_ uptake for the two measurements from each institution. We assume this slight difference is due to the difference in internal structures of two BILP-3 samples that were synthesized in two different batches independently at VCU and UW-Platteville. The BILPs are amorphous because of the irreversible nature of imidazole ring formation, and it is not surprising to obtain the final products with slightly different internal structures from two independently prepared samples. The BET surface areas and gas uptakes sometimes vary significantly, even for well-defined crystalline substances. For example, based on the synthetic methods, MOF-5 possesses a BET surface area in a wider range from 260 to 4400 m^2^ g^–1^.^36^

BILPs show a type-I isotherm, and desorption follows a moderate degree of hysteresis. A similar hysteresis pattern was observed for the cage molecule 6FT-RCC3, which has been attributed to the flexibility of polymers.^16,37^ In contrast to cage molecules, BILPs show no open loop hysteresis, which occurs due to swelling effects.^16,38^ The SO_2_ uptake of BILP-3 is 8.6 mmol g^–1^ (35 wt %) at 298 K and 1 bar as shown in Figure 2A. BILP-4 also shows a similar high SO_2_ uptake of 6.1 mmol g^–1^ (28 wt %) at the same temperature and pressure (Figure S2A).^39^ The sharp initial uptakes at low pressure are consistent with the expected high affinity of the imidazole moieties for SO_2_. At ambient pressure, the uptakes by BILPs exceed most porous materials of similar surface areas investigated to date (Table S1); FMOF-2 (2.2 mmol g^–1^ at 298 K),^40^ CoCo (2.5 mmol g^–1^ at 298 K),^41^ MFM-520 (3.38 mmol g^–1^ at 298 K),^42^ Pd(II)-based metal–organic case (6.0 mmol g^–1^),^17,43^ and rival the best-performing MOFs: NOTT-202a (∼8 mmol g^–1^ at 293 K), NOTT-300 (8.1 mmol g^–1^ at 273 K),^44^ and FM-300-(In) (8.28 mmol g^–1^ at 298 K)^45^ even though some of these MOFs have much higher surface area (NOTT-202; SA_BET_ = 2220 m^2^ g^–1^).^14,40^ The highest SO_2_ uptake of 25.7 mmol g^–1^ at 293 K and 1.0 bar was reported for MOF-177 (4100 m^2^ g^–1^).^20^ The SO_2_ uptake by BILP-3 at 298 K and 1 bar is comparable to TAM-POF (9.45 mmol g^–1^)^46^ and Viologen-POF (11.3 mmol g^–1^)^47^ and higher than the functionalized PIMs (PIM-1-AX: 7.32 mmol g^–1^, PIM-1: 5.89 mmol g^–1^, and PIM-1-COOH: 5.53 mmol g^–1^)^24^ and sPANs (sPAN-1: 5.56, sPAN-1: 5.64 mmol g^–1^).^23^ The storage capacity of BILPs also surpasses many of the organic solvents and ionic liquids^9^ and cage molecule CC3 (2.78 mmol g^–1^).^16^ The uptake by BILPs is somewhat lower than that of the KOH-treated benzimidazole-derived carbons such as BIDC-700 (10.25 mmol g^–1^) and BIDC-800 with (21.42 mmol g^–1^)^12^ and the best-performing cage molecule 6FT-RCC3 (13.78 mmol g^–1^).^16^ It is worth noting that BILP-3 possesses a significant SO_2_ uptake (4.5 mmol g^–1^) at low pressure (0.1 bar) relevant for removing SO_2_ trace contaminants from gas mixtures. This uptake is lower than the best-performing cage molecule 6FT-RCC3 (8.67 mmol g^–1^),^16^ MOF MIL-125(Ti)-NH_2_ (7.9 mmol g^–1^),^20^ and COF CTF-CSU41 (6.7 mmol g^–1^ at 0.15 bar)^48^ but higher than many other porous networks: MFM-170 (≈6.5 mmol g^–1^);^49^ MOF-177 (1.0 mmol g^–1^);^20^ CTF-CSU38 (4.4 mmol g^–1^ at 0.15 bar);^48^ P(Ph-4MVIm-Br) (4.14 mmol g^–1^).^50^

To understand the high SO_2_ uptake, the heat of adsorption (*Q*_st_) at zero coverage for BILP-3 has been calculated from experimental SO_2_ isotherms collected at 288, 298, and 308 K. The zero coverage *Q*_st_ is 32.0 kJ mol^–1^, which increases with SO_2_ uptake to a maximum of 38.3 and then drops to 25.0 kJ mol^–1^ at 1.0 bar (Figure 2C). This value is similar to that reported for SO_2_ interaction with NOTT-202a (35 kJ mol^–1^)^14^ and viologen-POF (38.3 kJ mol^–1^).^47^ The *Q*_st_ at zero coverage is lower than the *Q*_st_ value reported for the cage molecule with a secondary amine (RCC3: 82.78 kJ mol^–1^) but similar to those case molecules with a tertiary amine (6FT-RCC3: 43.03 kJ mol^–1^) and imine nitrogen (38.46 kJ mol^–1^).^16^ The high *Q*_st_ value for case molecule RCC3 suggests an almost irreversible chemisorption process,^16^ while the moderate *Q*_st_ for BILP-3 in this work suggests that it has a preference to undergo a physisorption-type interaction with SO_2_. Similar heat of adsorption values (29.2 and 32.3 kJ mol^–1^) were observed for cyanide (CN^–^) containing ionic liquids [N(CN)_2_]^−^-SO_2_^51^ and [C(CN)_3_]^−^-SO_2_, respectively.^52^ Significantly higher heat of enthalpy was reported for ionic liquids that contain thiocyanate (SCN^–^) anions: [SCN]^−^-SO_2_ (73.0 kJ mol^–1^), which is due to chemisorption of SO_2_.^52^ SO_2_ interacts chemically with the metal centers in some MOFs that also possess very high *Q*_st_ values, for example, Mg-MOF-74 (>90 kJ mol^–1^) and Zn-MOF-74 (>70 kJ mol^–1^).^53^ Moderate *Q*_st_ (35.83 and 28.39 kJ mol^–1^) has been reported for MOF-Th-Co-67 and MOF-Th-Co-66, respectively.^54^

To understand the reusability and stability of polymers upon exposure to SO_2_, BILPs were regenerated both at room temperature and at elevated temperature. For the regeneration processes (done at Northwestern University), BILP-3 was evacuated under vacuum at room temperature for 2 h prior to each adsorption data collection up to 1.0 bar. SO_2_ uptake for BILP-3 was recorded for three cycles, as shown in Figure 2B. The SO_2_ uptake slightly drops from the first cycle to the second cycle. However, it does not drop at all in the third cycle rather than slightly increases (BILP-3: 8.60, 8.12, and 8.29 mmol g^–1^). Almost identical SO_2_ uptakes suggest that BILPs can be regenerated at room temperature and are reusable. Repeated uptake measurements were also done at the PNNL for both BILP-3 and BILP-4. BILPs were activated at an elevated temperature (150 °C under vacuum), and the volumetric adsorption experiments were repeated for activated samples. The isotherms for three cycles for both BILPs are depicted in Figure S2B, which confirm the same uptake tendency (BILP-3: 7.23, 6.63, and 6.64 mmol g^–1^; BILP-4: 6.04, 5.87, and 6.10 mmol g^–1^). The slight fluctuation in the SO_2_ uptake during cycling is probably due to the flexible nature of the polymers, as stated earlier. A similar SO_2_ uptake pattern was reported for viologen-POF,^47^ BIDC,^12^ TAM-POF,^46^ and nanofiber aerogel.^55^ It is noteworthy to mention that BILPs are very robust under acidic and basic conditions. The synthesized BILPs were washed with 2.0 M HCl and NaOH. Therefore, we conclude that BILPs do not undergo any degradation or permanent chemical change under exposure to acidic SO_2_. The XPS spectra (discussed below) also preclude the chemisorption of SO_2_ by BILPs. These repeated SO_2_ uptake measurements thus suggest that BILPs are stable in SO_2_ environment and, can be regenerated at room temperature with the same efficiency as at elevated temperatures and can be reused effectively in SO_2_ capture. This result is very promising in terms of real-life applications of BILPs in SO_2_ capture compared to ionic liquids, which require high temperatures to regenerate the adsorbents.^9,56^

The chemical and thermal stability of BILPs after exposure to SO_2_ was also confirmed by TGA traces (Figure S3), which demonstrate two distinct weight losses. The initial weight loss at room temperature is due to the desorption of weak physisorbed SO_2_, while the second step started at ca. 200 °C and is proposed to be due to the desorption of strong physisorbed SO_2_. The strong physisorption is supported by the observation of a gradual weight loss upon heating to high temperatures. The total weight loss up to 300 °C is ∼30 wt %, which matches well with the SO_2_ uptake obtained from the adsorption isotherms. No significant weight loss was observed except desorption of the above calculated amount of adsorbed SO_2_, which demonstrates the stability of BILPs under SO_2_ adsorption environments. Although desorption of strongly physisorbed SO_2_ required high temperatures, it could be facilitated at lower temperatures under a vacuum, as observed in repeated SO_2_ adsorption–desorption experiments.

### Spectroscopic Characterization

The presence of adsorbed SO_2_ on BILPs was examined by FT-IR and XPS spectroscopy. The FT-IR spectra were recorded for BILPs before and after exposure of samples to SO_2_ (Figure S4). New sharp infrared (IR) peaks were observed at 1250 and 1120 cm^–1^ for SO_2_-dosed samples (BILPs@*n*SO_2_) and were assigned to asymmetric and symmetric stretching vibrational modes of adsorbed SO_2_, respectively. These two vibrational modes for gas phase free SO_2_ are observed at 1351 and 1147 cm^–1^, respectively.^57,58^ The lower frequency shifts of adsorbed SO_2_ compared to free SO_2_ arise from strong interactions of SO_2_ molecules with BILPs. It should be noted that larger downshifts, 1230 and 957 cm^–1^, were observed for chemically adsorbed SO_2_ in ionic liquids through sulfate formation (S=O and S–O stretches, respectively).^59^ An intense band appearing at 617 cm^–1^ is assigned to the bending mode of adsorbed SO_2_. A similar bending mode (624 cm^–1^) was reported for adsorbed SO_2_ onto tertiary amine-based Merrifield resins.^60^ The significantly higher frequency shift of the bending mode compared to isolated gas phase SO_2_ (508–518 cm^–1^)^61^ was explained by a noncovalent charge transfer between SO_2_ and the tertiary amine group.^60^

**Figure 3:**
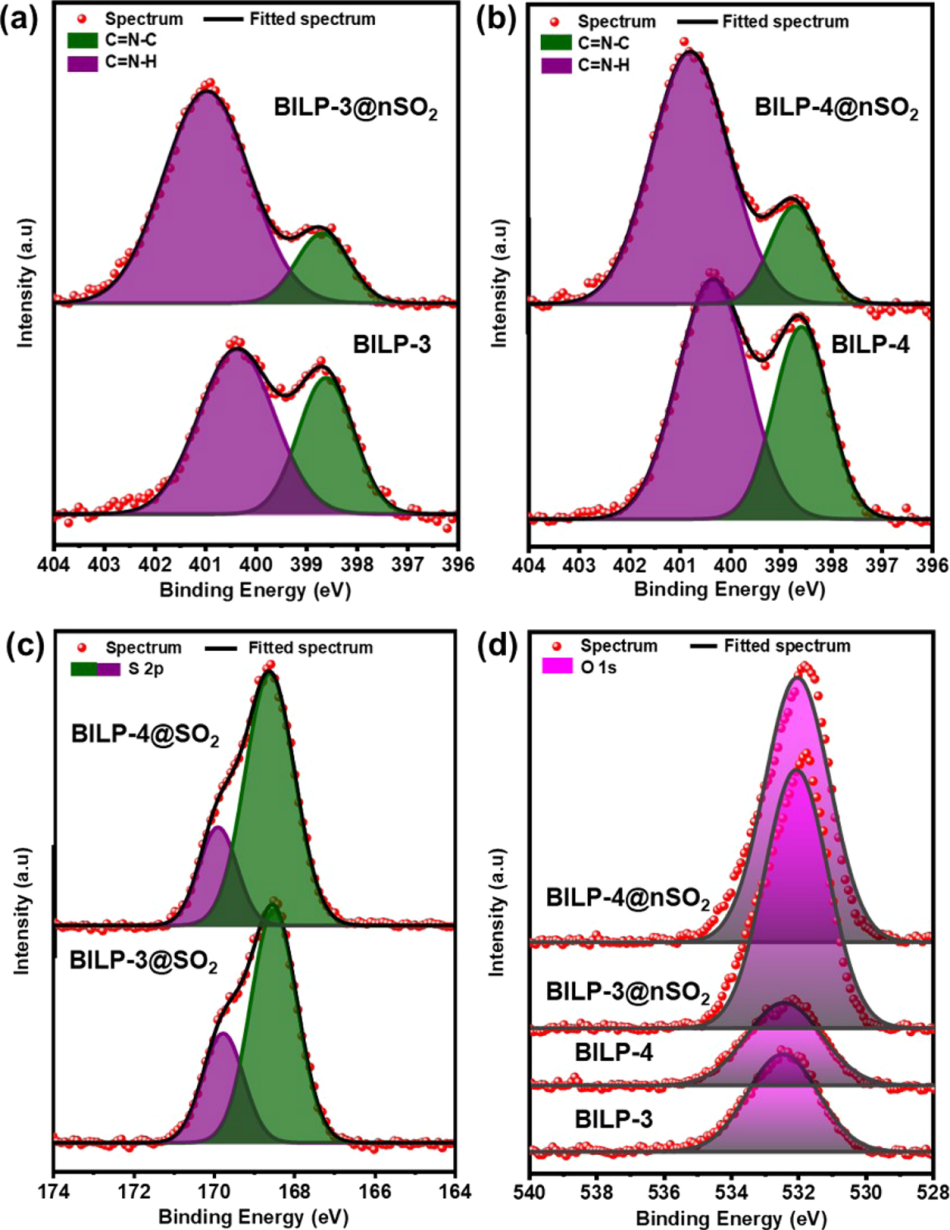
High-resolution XPS spectra for BILPs and BILPs@*n*SO_2_: (a) N 1s for BILP-3 and BILP-3@*n*SO_2_; (b) N 1s for BILP-4 and BILP-4@*n*SO_2_; (c) S 2p_3/2_ and S 2p_1/2_ for BILP-3@*n*SO_2_ and BILP-4@*n*SO_2_; (d) O 1s for BILP-3, BILP-4, BILP-3@*n*SO_2_, and BILP-4@*n*SO_2_.

XPS measurements were also used to explore the presence of adsorbed SO_2_ on the BILPs. Figure S5 shows the survey XPS spectra for BILPs before and after exposure to SO_2_. Detailed regional scans and deconvoluted peaks of S 2p, N 1s, and O 1s are shown in Figure 3. The intense C 1s peak at 284.1 eV in each spectrum (Figure S5) confirms the presence of aromatic carbons (sp^2^ carbon).^62^ The shoulder peak at 285.4 eV is due to C 1s in CN, and the trace at 289.8 eV is due to C 1s in residual carbonyl carbons (from unreacted aldehyde).^63−66^ The presence of the O 1s at around 532 eV in BILPs (Figure 3d) before exposure to SO_2_ most likely originates from unreacted aldehyde functional groups on the BILP surface. However, there is a significant intensity increase of the O 1s peak in BILPs@*n*SO_2_ compared to BILPs. This indicates significant adsorption of SO_2_ onto BILPs. The doublet pattern (398.6 and 400.4 eV) of the N 1s peak in BILPs (Figure 3a,b) with almost equal intensities was observed, which is consistent with the presence of two nitrogen sites with two different chemical environments in benzimidazole moieties: one is imine-type double-bonded nitrogen and the other is single-bonded nitrogen called pyridinic and pyrrolic nitrogen, respectively.^63^ Upon exposure to SO_2_, these peaks shift slightly to higher energy (398.7 and 400.9 eV), but the relative peak intensity of pyridinic nitrogen significantly decreases compared to that of pyrrolic nitrogen. This intensity alteration indicates that the pyridinic nitrogen in the benzimidazole ring is more affected by the adsorbed SO_2_. New peaks at around 169 and 232 eV were observed, which are assigned to S 2p and S 2s, respectively. These peaks confirmed the presence of adsorbed SO_2_ molecules in BILPs@*n*SO_2_.^67−69^ The presence of the S 2p_3/2_ peak at 168.6 eV (Figure 3c) confirms the physisorption of SO_2_ on BILPs. It has been reported that for multilayer physisorbed SO_2_, the S 2p_3/2_ values are within 167.6–169.4 eV, while for chemisorbed SO_2_, S 2p_3/2_ values are within 163.0–165.5 eV.^70−74^

### Theoretical Calculations

To understand the interaction between SO_2_ and BILPs, we carried out calculations based on DFT methods. Figure S6 shows the electrostatic potential surface of optimized structures for the benzimidazole-containing moieties of BILPs and SO_2_. As expected, the electronegative regions appear around the N-imine sites rather than those of N–H. A significant amount of electronegative charge is observed around the aromatic cores that appears to enhance the site-selective adsorption of polar SO_2_ gas molecules on benzimidazole-derived surfaces.

**Figure 4:**
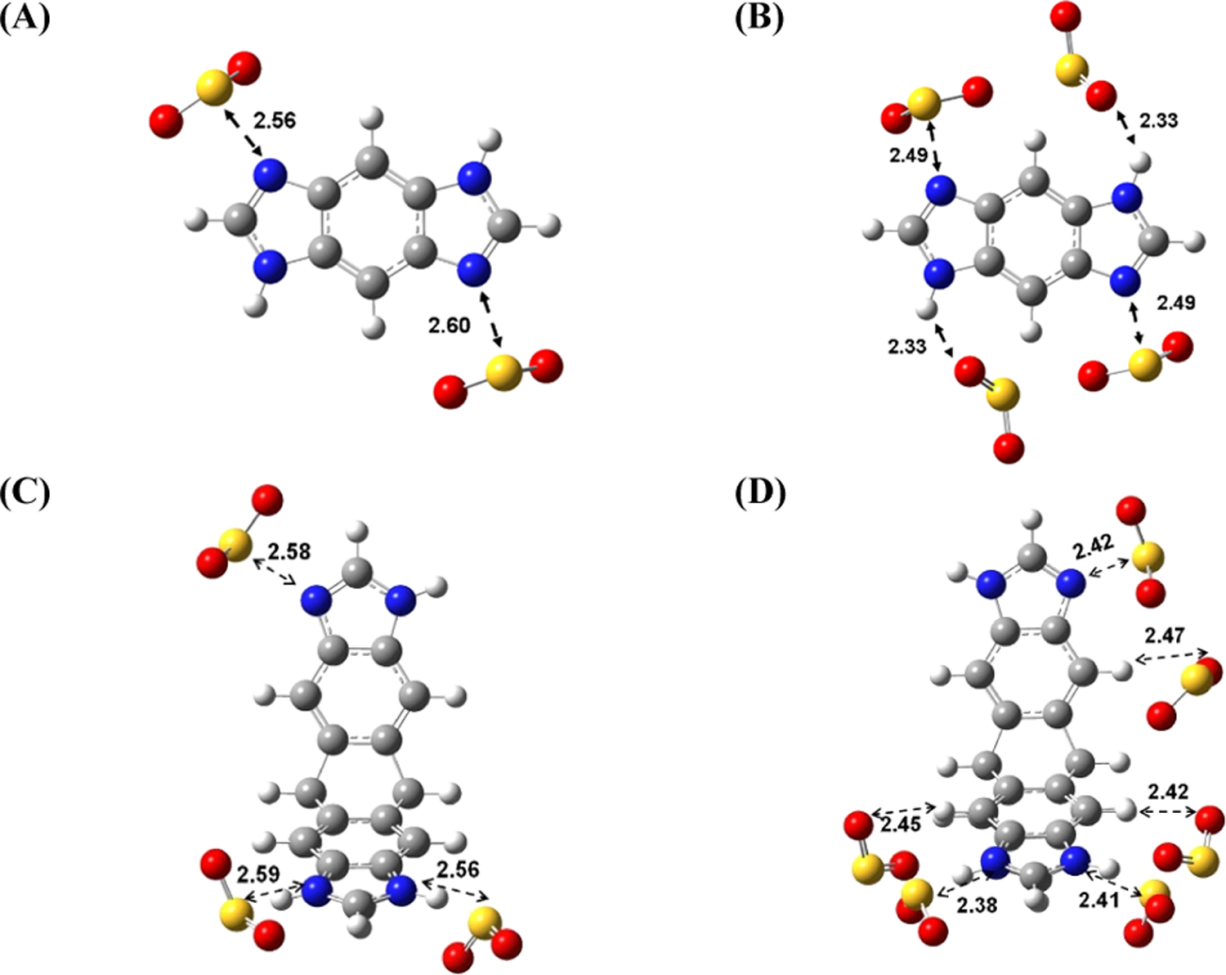
Optimized geometries of BILPs@*n*SO_2_ by using M06/6-311+G*. (A) BILP-4@2SO_2_, (B) BILP-4@4SO_2_, (C) BILP-3@3SO_2_, and (D) BILP-3@6SO_2_. BILP-4@*n*SO_2_ was fully optimized, while BILP-3@nSO_2_ was partially optimized for a constrained structure. Carbon is gray, nitrogen is blue, oxygen is red, sulfur is yellow, and hydrogen is white. The unit of bond length is angstrom (Å).

Two geometries were taken into consideration for BILPs@*n*SO_2_. One geometry considers a single SO_2_ molecule per imidazole ring (*n* = 1), and the other considers two SO_2_ molecules per imidazole ring (*n* = 2). For simplification, calculations were performed for the segments of BILP-3 and BILP-4 with three and two imidazole rings, respectively. In BILP-3, imidazole rings are well separated by the triptycene moiety, while in BILP-4, two imidazole rings are relatively closely separated by the benzene ring. Calculated results on BILP-4 are discussed first and then compared to BILP-3. Fully optimized geometries for BILP-4@*n*SO_2_ using M06/6-311+G* are shown in Figure 4A,B. SO_2_ molecules preferably lie close to the N-imine site when two SO_2_ molecules were allowed to interact with BILP-4, with the distance between the N-imine site and SO_2_ molecule being about 2.58 Å (*d*_SN_) (Figure 4A). On the other hand, when four SO_2_ molecules interact, two of them (called SO_2_(I)) lie close to the N-imine site with a distance of *d*_SN_ = 2.49 Å, whereas the other two SO_2_ molecules (called SO_2_(II)) lie in between aryl-H and N–H of imidazole rings (Figure 4B). To understand the bonding preference of SO_2_ for N-imine and N–H of the imidazole ring, we examined the NBO charges on the interacting atoms. The NBO charges on the S atom (+1.62e) of SO_2_ and N-imine (−0.56e) of the imidazole ring indicate the presence of a strong dipole–dipole interaction. The NBO charges on O atom (−0.86e) of SO_2_ and the atom –H (+0.44e) of N–H indicate a relatively weaker hydrogen bonding. This suggests the preferential adsorption of SO_2_ on N-imine of the imidazole ring.

The orientation of SO_2_(II) is such that the S atom is directed away from the H atom of the six-membered aryl ring (*d*_SH_ = 3.13 Å) and is closer to the O atom of SO_2_(I) (*d*_OS_ = 2.76 Å). On the other hand, one of the O atoms of SO_2_(II) is directed toward the H atom of N–H (*d*_OH_ = 2.33 Å), while its distance from the H atom of aryl-H is 2.48 Å. This orientation of SO_2_(II) suggests an intermolecular attraction between the O atom of SO_2_(I) and the S atom of SO_2_(II) that slightly displaces the SO_2_ molecules from the BILP-plane (Figure S7). Similar SO_2_–SO_2_ interactions with an approximate intermolecular distance of 3.13 Å have been reported for MOF MFM-250.^42^ Geometry optimization was also performed using LDA/6-311+G*, and the optimized geometries of BILP-4@*n*SO_2_ (Figure S8) showed a similar spatial orientation of SO_2_ molecules, though the LDA method showed closer proximity to the adsorption sites. This is because the LDA method^32^ overestimates binding.

Inspection of the electrostatic potential energy surface, as shown in Figure S6, revealed that the six-membered aryl ring possesses a significant electronegative charge that creates a partial positive charge on the attached hydrogen (aryl-H(δ^+^)). This may attract a partially negative O atom of SO_2_(II). To understand whether the aryl-H(δ^+^) of six-membered rings or the N–H(δ+) of the imidazole ring has more attraction to (δ^–^)O=S=O(II), calculations were performed for BILP-3 in which the imidazole moieties are far apart, allowing a better understanding of the above effects.

The geometries of BILP-3@*n*SO_2_ were optimized using both the LDA and M06 level of theory for a segment of polymer that contains the triptycene core. It should be noted that the interaction of six SO_2_ molecules with BILP-3 results in bending or distortion of the BILP-3 strand when the geometry is fully optimized by using either LDA or M06. The distortion is relatively large for the LDA structure (Figure S9). Although this distortion for an isolated segment in the presence of SO_2_ molecules may be explained in terms of strong intermolecular forces, such a large distortion in a real extended polymer network is unlikely. Therefore, a partial optimization was performed for a constrained structure of BILP-3@*n*SO_2_ in which the polymer segment was frozen, and the binding sites of SO_2_ molecules were only optimized (Figures S10 and S11). Figure 4C,D shows the partially optimized structures of BILP-3@3SO_2_ and BILP-3@6SO_2_ using the M06/6-311+G* level of theory. As expected, SO_2_ molecules in BILP-3@3SO_2_ preferably lie closer to the N-imine sites of the imidazole rings with an average sulfur–nitrogen distance of 2.58 Å, the same as that observed in fully optimized BILP-4@2SO_2_. When six SO_2_ molecules interact in BILP-3@6SO_2_, three of them preferably bind to the N-imine sites, as expected, with an average sulfur–nitrogen distance of 2.42 Å. The remaining three SO_2_ molecules lie close to the H atom of aryl-H that is located at the N-imine sites rather than the N–H site of imidazole moieties (Figure 4D). This result was surprising because it was expected that the later three SO_2_ molecules were likely to lie toward the N–H sites. This spatial localization of SO_2_ molecules can be explained by combining the effect of a higher electronegative charge around the benzene ring, which creates aryl-H partially positive (aryl-H(δ^+^)···O(δ^–^)=S=O) and the cooperativity between SO_2_(I) and SO_2_(II) molecules (intermolecular O=S=O(δ^–^)···S(δ^+^)O_2_). This combined attractive force appears to overcome the attraction between SO_2_(II) and N–H of the imidazole ring (O=S=O(δ^–^)···H(δ^+^)N). However, the attractive force between SO_2_(II) and N–H of the imidazole ring in BILP-4@4SO_2_ works together with the other two combined forces because all are closely spaced on the same side. This is supported by the fact that the binding energy (BE) of BILP-4@4SO_2_ (42.31 kJ mol^–1^) is slightly higher than that of BILP-3@6SO_2_ (40.31 kJ mol^–1^, Table 1). The calculated binding energy for BILP-3@6SO_2_ (40.31 kJ mol^–1^) using M06 supports the experimental heat of adsorption. Experimental *Q*_st_ for BILP-3 at zero coverage, namely, 32.0 kJ mol^–1^, increases with SO_2_ uptake to a maximum of 38.3 and then gradually drops to 25.0 kJ mol^–1^ at 1.0 bar (Figure 2C). The increase in the *Q*_st_ value with the increasing SO_2_ uptake appears to be due to the cooperative nature of the adsorbed SO_2_ molecules. Similar guest–guest interaction between two adjacent SO_2_ molecules has been reported for MOF MFM-520 and for fluorinated anion-pillared metal–organic frameworks (APMOFs).^42,75^ Higher binding energy in LDA compared to M06 level of theory as summarized in Table 1 accounts for the overestimation of the binding in the LDA method.^32^ Our experimental and calculated binding energies from the M06 level of calculations are in good agreement with the binding energy reported for imidazole (39.1 kJ mol^–1^) by Shannon et al.^76^ A similar result (40.52 kJ mol^–1^) was reported for SO_2_–tetrazine interaction based on the Monte Carlo simulation.^77^ Other reports on SO_2_ capture by azole-based ionic liquids, derived from tetrazole and imidazole moieties, demonstrated that the electronegative nitrogen atoms of the anions can modulate bonding strength and lead to high absorption of SO_2_.^78^ The reported calculated enthalpies for SO_2_–tetrazole complexes were 89.3, 59.9, 39.7, and 34.4 kJ mol^–1^ for [Tetz]@SO_2_, [Tetz]@2SO_2_, [Tetz]@3SO_2_, and [Tetz]@4SO_2_, respectively. Similar results were also reported for the SO_2_–imidazole interactions [Im]@SO_2_ (124.6 kJ mol^–1^), [Im]@2SO_2_ (75.7 kJ mol^–1^), [Im]@3SO_2_ (36.9 kJ mol^–1^), and [Im]@4SO_2_ (30.3 kJ mol^–1^). The gradual decrease in enthalpies in these series was explained by the change of interactions from chemical to physical types.^78^ The closest calculated binding energy among the cage molecules is for CC3 (49.7 kJ mol^–1^), which contains imine nitrogen, while the other case molecules have significantly higher binding energies (86.4 kJ mol^–1^ for RCC3, which contains a secondary amine; 68.6 kJ mol^–1^ for FT-RCC3, which contains tertiary amine).^16^ Higher binding energy for amine (−NH_2_) functionalized MOF NH_2_-MIL-53(AL) was reported to be 67.3 kJ mol^–1^, which is dominated by the strong interactions of SO_2_ with two NH_2_ groups from two different directions (N–H(δ^+^)···(δ^–^)OSO and O_2_S(δ^+^)···(δ^–^)N–H).^79^ Based on the above discussions, it is expected that BILPs, which have neutral imidazole moieties in their backbones, exhibit only physisorption interactions with SO_2_. The calculated binding energy using the M06 method for BILPs@*n*SO_2_ not only supports the physisorption assumptions but also supports the superiority of M06 over LDA at least when the adsorption is physisorption type.

**Table 1:** Calculated Binding Energies (BE) of BILPs@*n*SO_2_ Using the LDA and M06 Methods

	BE	
BILPs@*n*SO_2_	LDA/6-311+G*	M06/6-311+G*
BILP-4@2SO_2_	–73.33 kJ mol^–1^	–42.01 kJ mol^–1^
BILP-4@4SO_2_	–72.36 kJ mol^–1^	–42.31 kJ mol^–1^
BILP-3@3SO_2_	–71.91 kJ mol^–1^	–41.94 kJ mol^–1^
BILP-3@6SO_2_	–67.80 kJ mol^–1^	–40.31 kJ mol^–1^

### Selective Gas Adsorption

**Figure 5:**
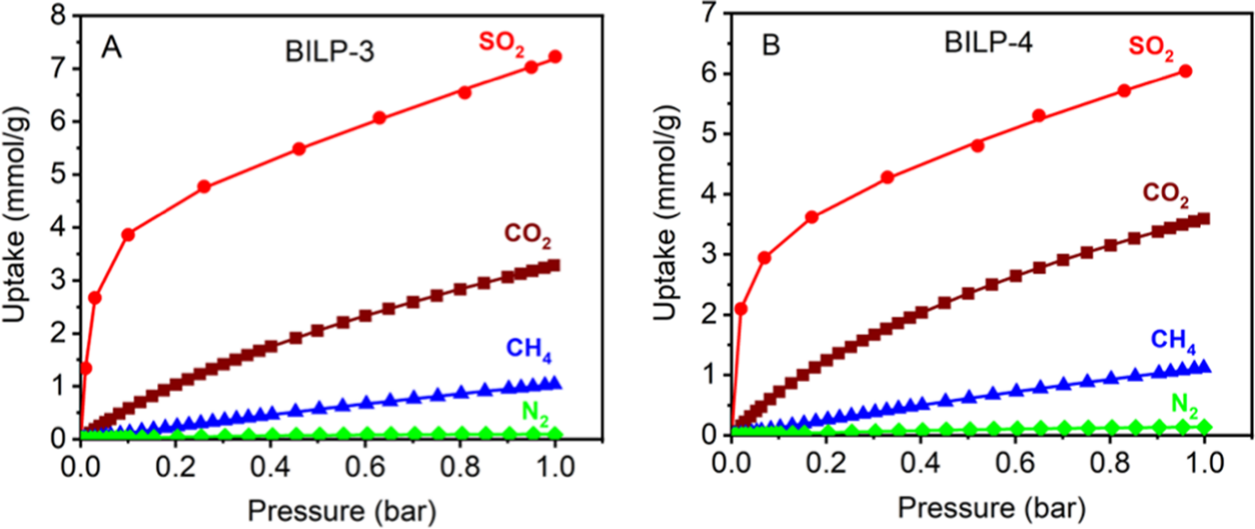
Low-pressure SO_2_, CO_2_, CH_4_, and N_2_ uptake capacities for (A) BILP-3, and (B) BILP-4 at 298 K. Solid markers in each plot represent the experimental data, while the solid smooth lines represent the fitted plots.

Because of its high toxicity, trace levels of SO_2_ must be captured before releasing flue gas or other SO_2_-containing gas mixtures into the atmosphere. Selective adsorption is essential for the effective capture and separation of trace levels of SO_2_ from gas mixtures. Our earlier reports demonstrated that BILPs have a significant storage capacity of CO_2_ and have very high CO_2_ adsorption selectivity over CH_4_ and N_2_.^26−28,80^ This encouraged us to evaluate the efficiency of BILPs in the selective capture of SO_2_ from common gas mixtures that contain trace levels of SO_2_. The adsorption isotherms were fitted to dual site for SO_2_, CO_2,_ and CH_4_ and single site for N_2_ using Langmuir Freundlich models (Figure 5). The fitted isotherms were used to calculate the selectivity based on the initial slope calculations (Figure S12). Significantly high SO_2_ uptake capacities of BILP-3 and BILP-4 were observed compared to our reported CO_2_, CH_4_, and N_2_ uptakes (Figures 5 and S12).^26,27^ Comparison of the gas adsorption isotherms and the initial slope calculations clearly demonstrated the superiority of BILPs in selective capture of SO_2_ (BILP-3: SO_2_/CO_2_, 24; SO_2_/CH_4_, 118; SO_2_/N_2_, 674; CO_2_/N_2_, 29; BILP-4: SO_2_/CO_2_, 19; SO_2_/CH_4_, 113; SO_2_/N_2_, 600; CO_2_/N_2_, 31). Although the adsorption of SO_2_ on BILPs has been attributed to the physisorption type as discussed in earlier sections, the relative adsorption affinity onto BILPs, which have basic nitrogen sites, is expected to be higher for SO_2_ than that for CO_2_ because of the higher acidic nature of SO_2_. This is further supported by their *Q*_st_ values. The *Q*_st_ values for CO_2_ and CH_4_ for BILP-3 at zero coverage are 28.6 and 16.6 kJ mol^–1^, respectively, and they decrease steadily with increasing the gas uptakes.^26^ A relatively higher *Q*_st_ value of 32.0 kJ mol^–1^ and the cooperative nature of adsorbed SO_2_ provide higher adsorption selectivity for SO_2_ over that of CO_2_ at low concentrations. Furthermore, SO_2_ is a polar molecule while CO_2_ is nonpolar, and this polarity difference plays the key role in the preferential adsorption of SO_2_ through dipole–dipole interaction. Nonpolar N_2_ as the inert gas has the lowest affinity in the adsorption scale. These findings indicate the potential of BILPs in selective SO_2_ removal from gas mixtures, where its concentration is very low.

## Conclusions

We demonstrated that BILPs show a very high uptake of acidic SO_2_ gas. These porous polymeric frameworks are regenerated easily and can be reused without losing their initial gas uptake capacity. The moderate heat of adsorption confirms relatively strong site-selective physisorption as predicted by DFT calculations. Initial slope calculations predict that the BILPs would be effective in the selective capture of SO_2_ at low concentrations from flue gas components.

## Supporting Information Available

Procedures for SO_2_ gas adsorption, TGA, FT-IR, XPS, gas uptake selectivity, and theoretical data (PDF)
