# Supplementary material for: High-Performance Porous Organic Polymers for Environmental Remediation of Toxic Gases
Source: Langmuir. 2024 Apr 4;40(15):8024–34. doi: 10.1021/acs.langmuir.3c03980 (PMC11025134; doi:10.1021/acs.langmuir.3c03980)
Supplement: Supplementary file 1 — la3c03980_si_001.pdf [file la3c03980_si_001.pdf]

Supporting Information

High-Performance Porous Organic Polymers for Environmental Remediation of Toxic Gases

Mohammad G. Rabbani[*], Riley K. Sasse[,†], Swayamprabha Behera[□], Puru Jena[‡], Jian Liu[▫], Praveen K. Thallapally[▫], Timur Islamoglu[♂], Mohammad K. Shehab[□], Mahmoud M. Kaid[§], Omar K. Farha[□], Hani M. El-Kaderi*[§]

Department of Chemistry, University of Wisconsin-Platteville, Platteville, WI-53818, USA

[†]Department of Physics, Kennesaw State University, Marietta Campus, 1100 South Marietta Pkwy, Marietta, GA 30060, USA

[□]Department of Physics, Virginia Commonwealth University, Richmond, VA-23284, USA

[▫]Pacific Northwest National Laboratory, Richland, Washington 99352, USA

[♂]UL Research Institutes, Materials Discovery Research Institute, 8045

Lamon Avenue, Skokie, Illinois 60077, USA

[□]Department of Chemistry, Northwestern University, 2145 Sheridan Road, Evanston, Illinois 60208, USA

[§]Department of Chemistry, Virginia Commonwealth University, Richmond, VA-23284, USA

[*]Mohammad G. Rabbani: rabbanim@uwplatt.edu

[*]Hani M. El-Kaderi: helkaderi@vcu.edu

Table of Contents

SO_2 Sorption Measurement **3**

SO_2 Sorption Isotherms **5**

SO_2 Sorption (Table S1) for Selected Porous Materials **6**

Thermogravimetric Analysis **7**

FT-IR Measurement **8**

XPS Measurement **9**

Computational Results **10-14**

Gas Adsorption Selectivity Studies **15**

References **15**

EXPERIMENTAL SECTION

Dynamic SO_2 sorption Measurement. The volumetric system used to measure SO_2 adsorption and desorption isotherms is shown in Figure S1. Chamber A is loaded with adsorbents and chamber B is used to dose and evacuate gas to and from chamber A. The volumes of chamber A and chamber B and the volumes of the tubes connecting them were determined before the experiment. Valve 1 and 2 were used to control the dosing and evacuating processes. Valve 3 is a three-way value which can be used to conveniently switch the system between adsorption and desorption measurements.

The apparatus was installed inside an environmental chamber to keep the temperature constant. The whole system is put inside a chemical hood for safety considerations. A powder sample (about 120 mg) was placed in chamber A (volume of VA plus the volume of tubes after V1 is 4.134 cm^3) with a layer of glass wool on top to prevent sudden suction of the powder sample. The sample was activated at 150 °C for 12 h with a dynamic vacuum before each measurement. Chambers A and B were evacuated through valve V3 with V2 and V1 open. Chamber A was then sealed under vacuum by closing V1. The pressures in A and B were recorded automatically using electronic transducers PA and PB (Wika ECO series) interfaced to a computer equipped with LabView. While activated sample is sealed in chamber A under vacuum, a known amount of SO_2 was introduced into chamber Band then V2 is shut. V1 is open to let the gas adsorption reach equilibrium. This step was repeated as necessary to obtain the adsorption data points. As for the desorption part, a similar but reverse procedure was adopted. The chamber B was evacuated to vacuum and then V1 was opened to let the gas desorption reach equilibrium. This step was also repeated as necessary to obtain desorption data points. The criterion for equilibrium is that the change of pressure is less than 0.1% within 15 min. The recorded pressures were used to calculate

the adsorbed amount based on basic mass balance equations. Ideal gas law was adopted and the volume of the sample was estimated to be 0.15 cm^3, which needs to be subtracted from the volume A during the calculation. The activation procedure as described before was repeated before proceeding to the next cycle of SO_2 adsorption and desorption measurement.

Figure S1. Dynamic sorption system for the SO_2 isotherm measurements.

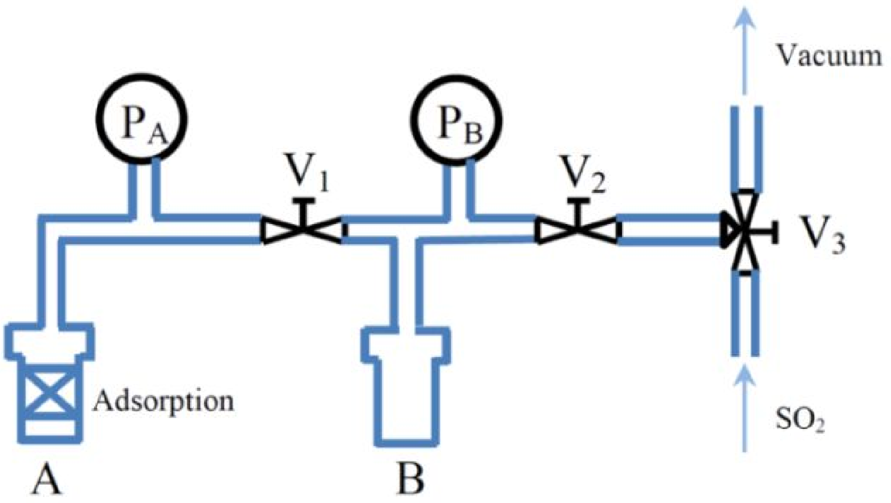

Figure S2: Dynamic SO_2 adsorption-desorption isotherms for BILP-3 (indicated by black color) and BILP-4 (indicated by red color) at 298 K (A). Filled (adsorption) and empty (desorption) data. Recyclability of SO_2 sorption for BILP-3 and BILP-4 at 298 K (B).

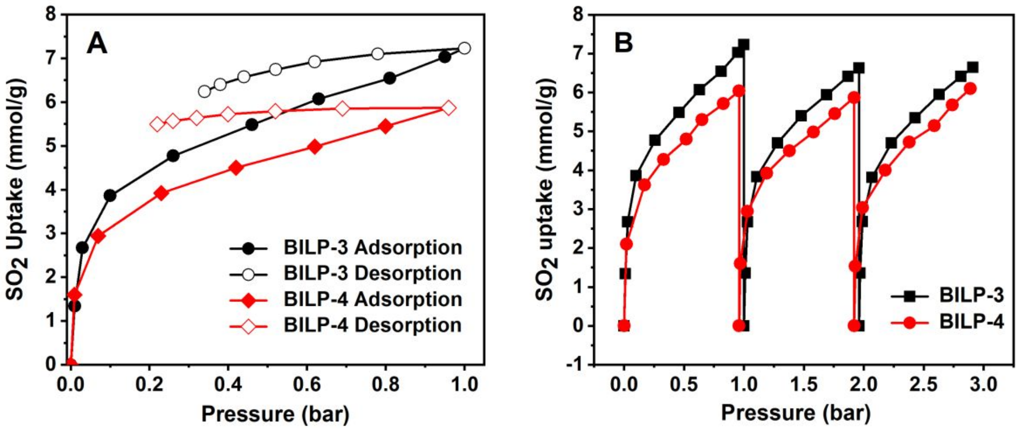

Table S1: SO_2 adsorption for selected materials.

Materials		Surface areas (BET)		Conditions		SO_2 uptake/ (mmol g^{-1})		References
BILP-3		1306 (m^2 g^{-1})		RT, 1 bar		7.2 (32%)		This work
BILP-4		1134 (m^2 g^{-1})		RT, 1 bar		6.1 (28%)		This work
MOF-5		2205 (m^2 g^{-1})		RT, 1 bar		0.016 (0.1%)		Ref[1]
IRMOF-3		1568 (m^2 g^{-1})		RT, 1 bar		0.094 (0.6%)		Ref [1]
MOF-74		632 (m^2 g^{-1})		RT, 1 bar		3.0 (16.2%)		Ref [1]
MOF-177		3875 (m^2 g^{-1})		RT, 1 bar		< 0.016 (<0.1%)		Ref [1]
MOF-199		1264 (m^2 g^{-1})		RT, 1 bar		0.50 (3.1%)		Ref [1]
IRMOF-62		1814 (m^2 g^{-1})		RT, 1 bar		< 0.016 (<0.1%)		Ref [1]
BPL Carbon				RT, 1 bar		0.52 (3.2%)		Ref [1]
FMOF-2		378 (m^2 g^{-1})		RT, 1 bar		2.2 (12.4%)		Ref [2]
CoCo		712 (m^2 g^{-1})		RT, 1 bar		2.5 mmol g^{-1} (13.8%)		Ref [3]
ZnCo		710 (m^2 g^{-1})		RT, 1 bar		1.8 mmol g^{-1} (10.3%)		Ref [3]
NOTT-202a		2220 (m^2 g^{-1})		293 K and 1 bar		~ 8 mmol g^{-1} (33.9%)		Ref [4]
NOTT-300		1370 (m^2 g^{-1})		273 K and 1.0 bar		8.1 mmol g^{-1} (34.2%)		Ref [5]
Pd(II)-MOFs		111 (m^2 g^{-1})		273 K and 1.0 bar		6.0 mmol g^{-1}		Ref[6]
FT-RCC3		396 (m^2 g^{-1})		298 K and 1 bar		13.78 mmol g^{-1}		Ref[7]
MFM-300-(In)		1100 (m^2 g^{-1})		298 K and 1 bar		8.28 mmol g^{-1}		Ref[8]
MOF-177		4050 (m^2 g^{-1})		293 K and 1 bar		25.7 mmol g^{-1}		Ref[9]

Thermogravimetric (TGA) analysis: TGA analysis was carried out using a TA Instruments Q-5000IR series thermal gravimetric analyzer with samples held in 50 μL platinum pans under an atmosphere of N_2 (heating rate 5 °C/min).

Figure S3. TGA traces for BILP-3@nSO_2 and BILP-4@nSO_2.

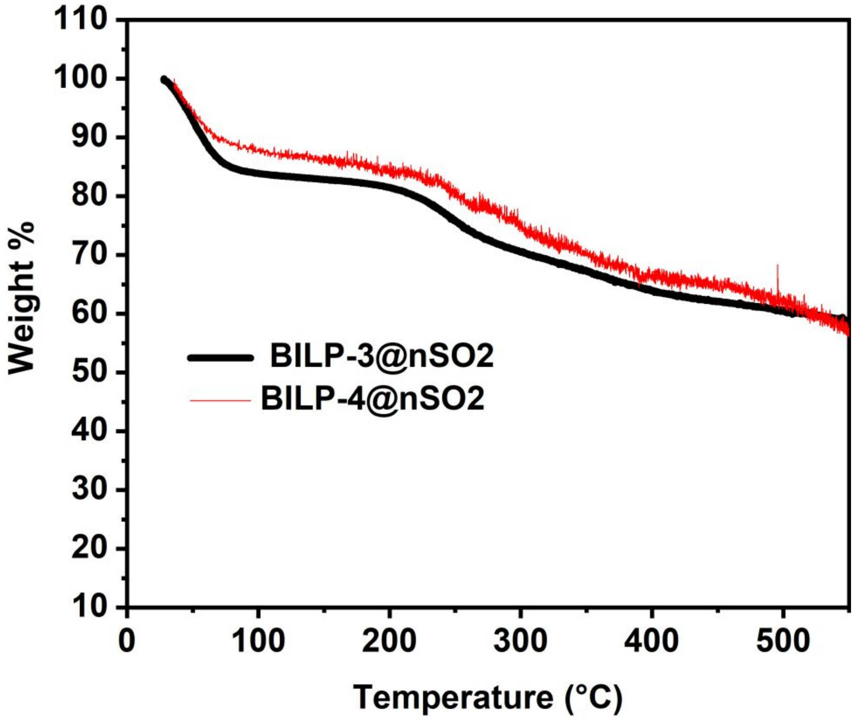

FT-IR Measurement: FT-IR spectra were obtained as KBr pellets using a Nicolet-Nexus 670 spectrometer.

Figure S4. FT-IR spectra for BILPs before and after dosed SO_2.

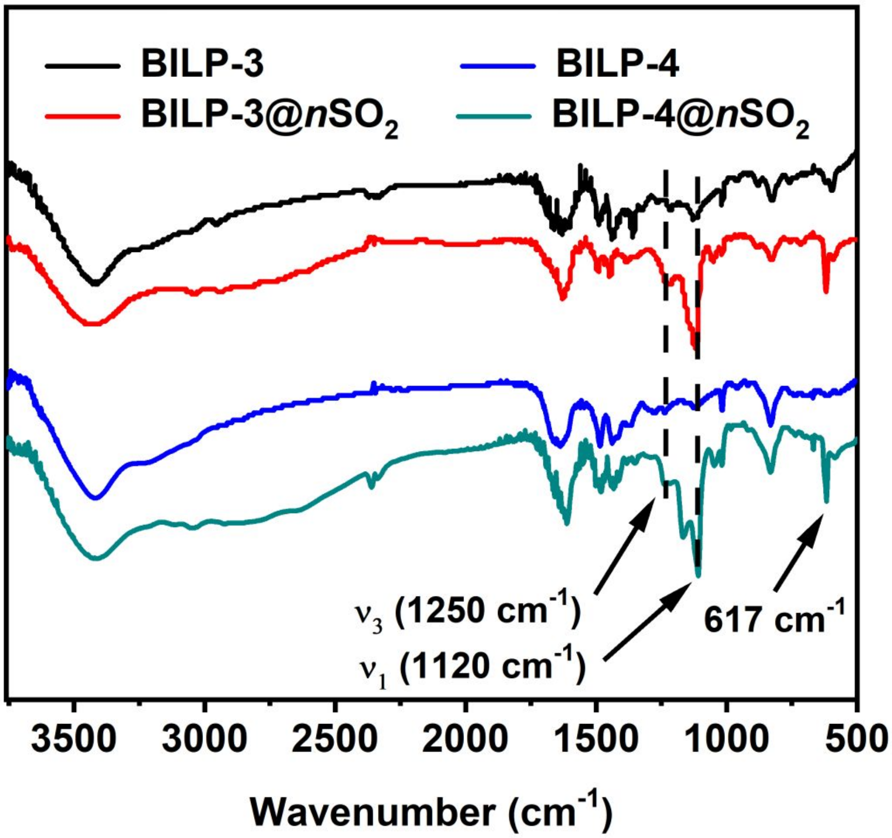

X-Ray Photoelectron Spectroscopy (XPS) measurement: XPS measurement was performed on a Thermo Scientific ESCALAB 250 microprobe with a focused monochromatic Al Ka x-ray (1486.6 eV) source and a 180 hemispherical analyzer with a six-element multichannel detector. Charge compensation was employed during data collection by using an internal flood gun (2 eV electrons) and a low-energy Ar^+ external flood gun.

Figure S5. a) XPS survey spectra for BILPs and BILPs@nSO_2; b) high-resolution XPS spectra of C 1s for BILP-3 and BILP-3@nSO_2; c) high-resolution XPS spectra of C 1s for BILP-4 and BILP-4@nSO_2.

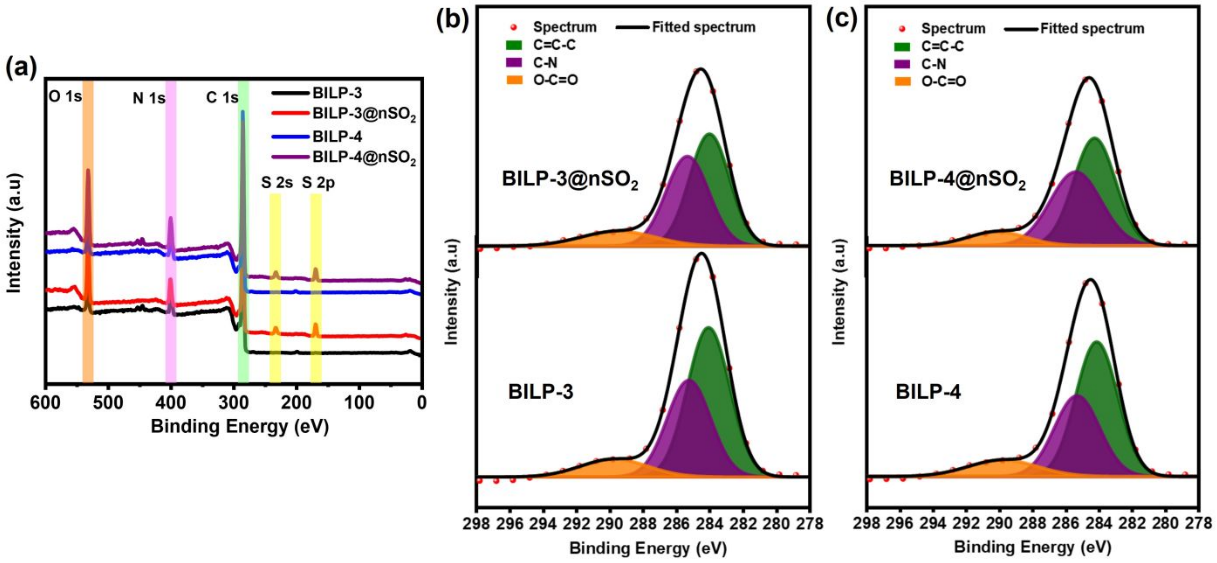

Table S2. XPS data for pristine BILPs and SO_2 adsorption studies.

Peaks	Binding energy (eV)			
	BILP-3	BILP-3@nSO_2	BILP-4	BILP-4@nSO_2
S2p	-	168.6, 169.9 (s)	-	168.5, 169.8 (s)
C1s	284.1, 285.3, 289.8	284.1, 285.4, 289.7	284.1, 285.4, 289.8	284.2, 285.4, 289.8
N1s	398.6, 400.4	398.7, 400.9	398.6, 400.4	398.7, 400.8
O1s	532.4	532.1	532.5	532.0

Figure S6. Electrostatic potential surfaces for core-segments of BILPs. a) Segment of BILP-4, b) segment of BILP-3 (front view), c) segment of BILP-3 (side-view), d) SO_2. Electrostatic potential surfaces were obtained from the geometry optimization at LDA/6-311+G* level of theory.

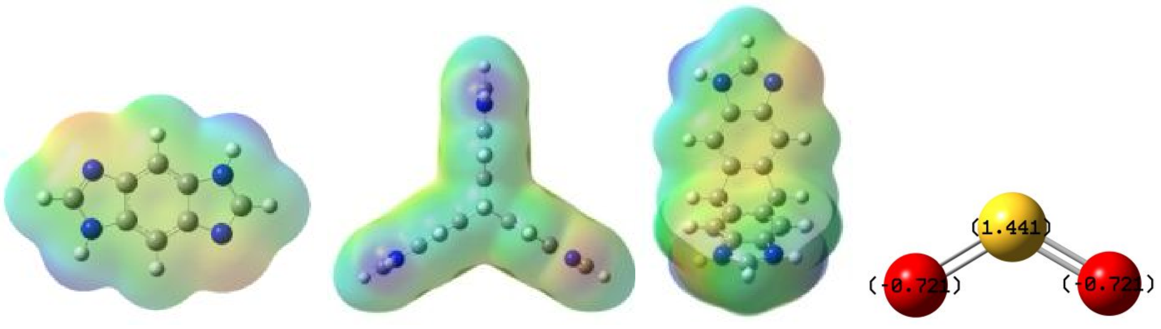

(a) **(b)** **(c)** **(d)**

Figure S7. Fully optimized geometries of (a) BILP-4@2SO_2 and (b) BILP-4@4SO_2. Side view for calculated at M06/6-311+G* level of theory. The blue, grey, white, yellow and red colors stand for N, C, H, S and O atoms, respectively.

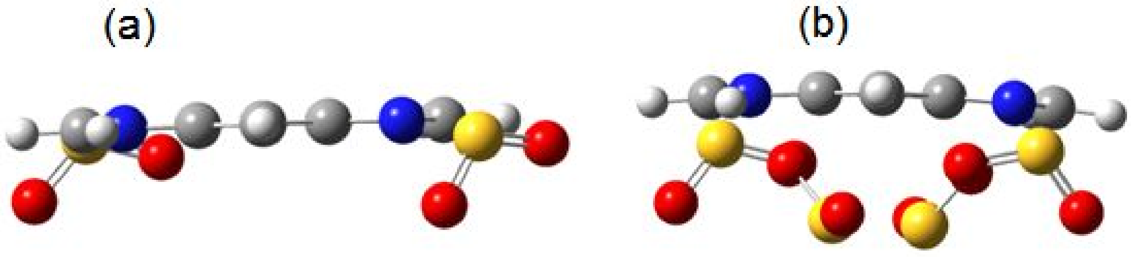

Figure S8. Fully optimized geometries of (a) BILP-4@2SO_2 and (b) BILP-4@4SO_2 calculated at LDA/6-311+G* level of theory. The bond lengths are in Å. The blue, grey, white, yellow and red colors stand for N, C, H, S and O atoms, respectively.

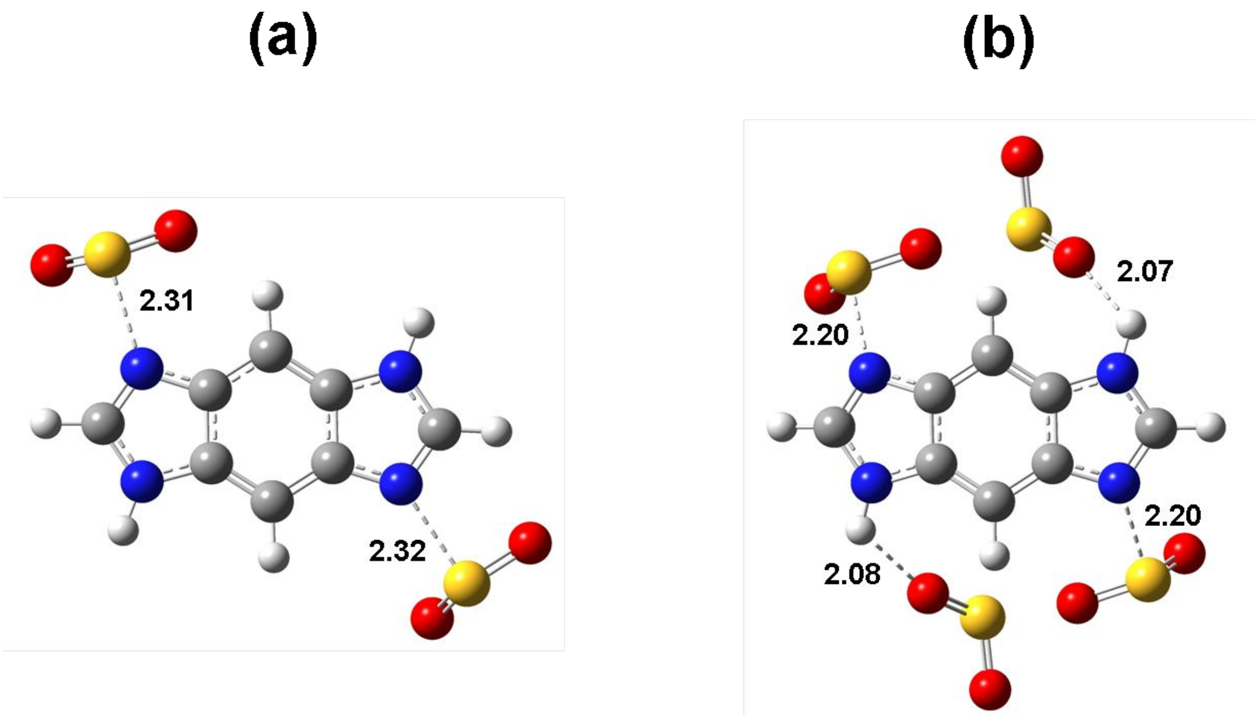

Figure S9: Fully Optimized geometries of BILP-3@6SO_2 calculated at (a) LDA/6-311+G* and (b) M06/6-311+G* level of theory. The blue, grey, white, yellow and red colors stand for N, C, H, S and O atoms.

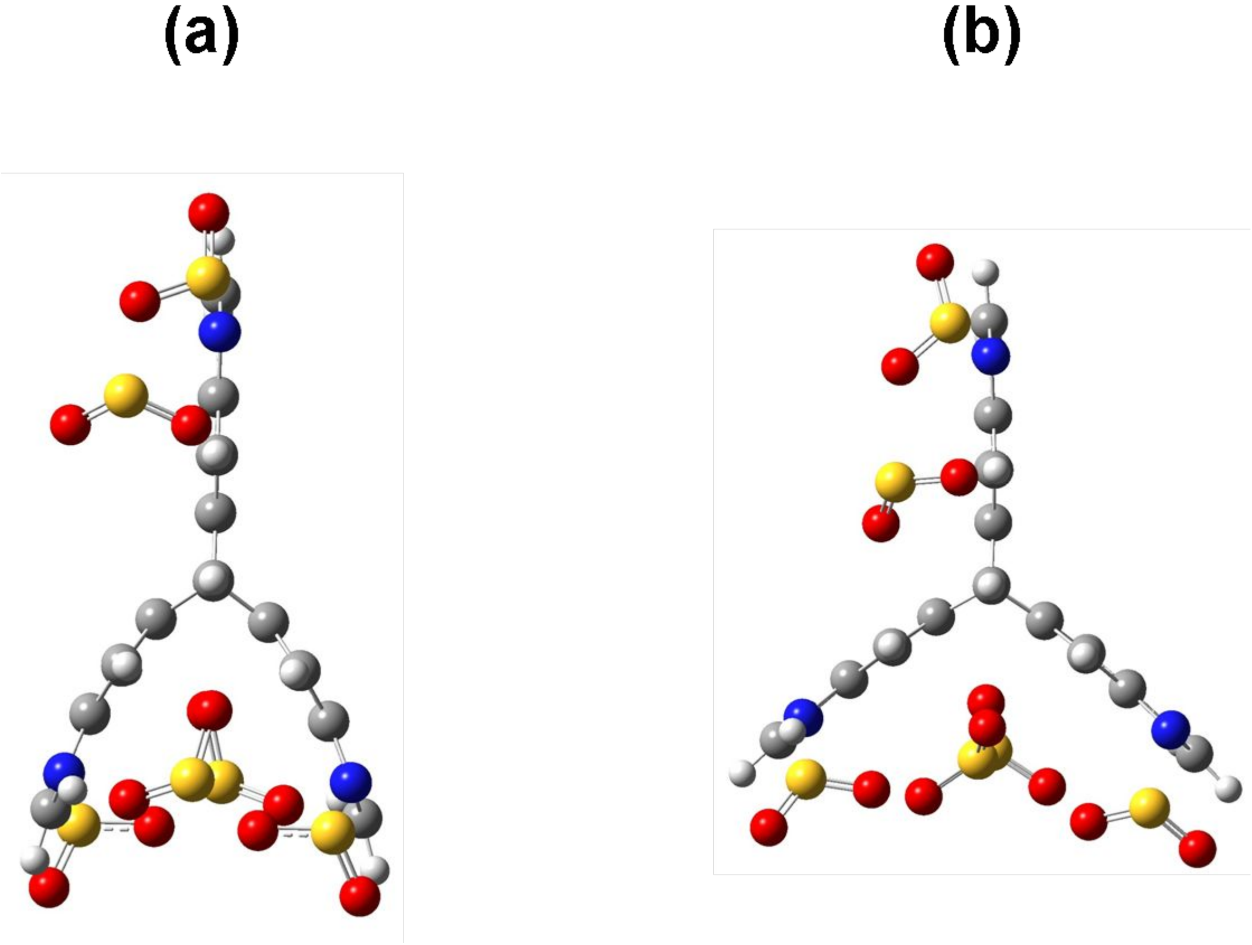

Figure S10. Partially optimized Geometries of (a) BILP-3@3SO_2 and (b) BILP-3@6SO_2 calculated at M06/6-311+G* level of theory. These were obtained by carrying out constrained optimization of BILP-3@nSO_2, where we froze the BILP-3 segment and only allowed optimization of binding sites of the SO_2 molecules. The bond lengths are in Å. Front and side views are given on the left and right panel, respectively. The blue, grey, white, yellow and red colors stand for N, C, H, S and O atoms.

(a)

(b)

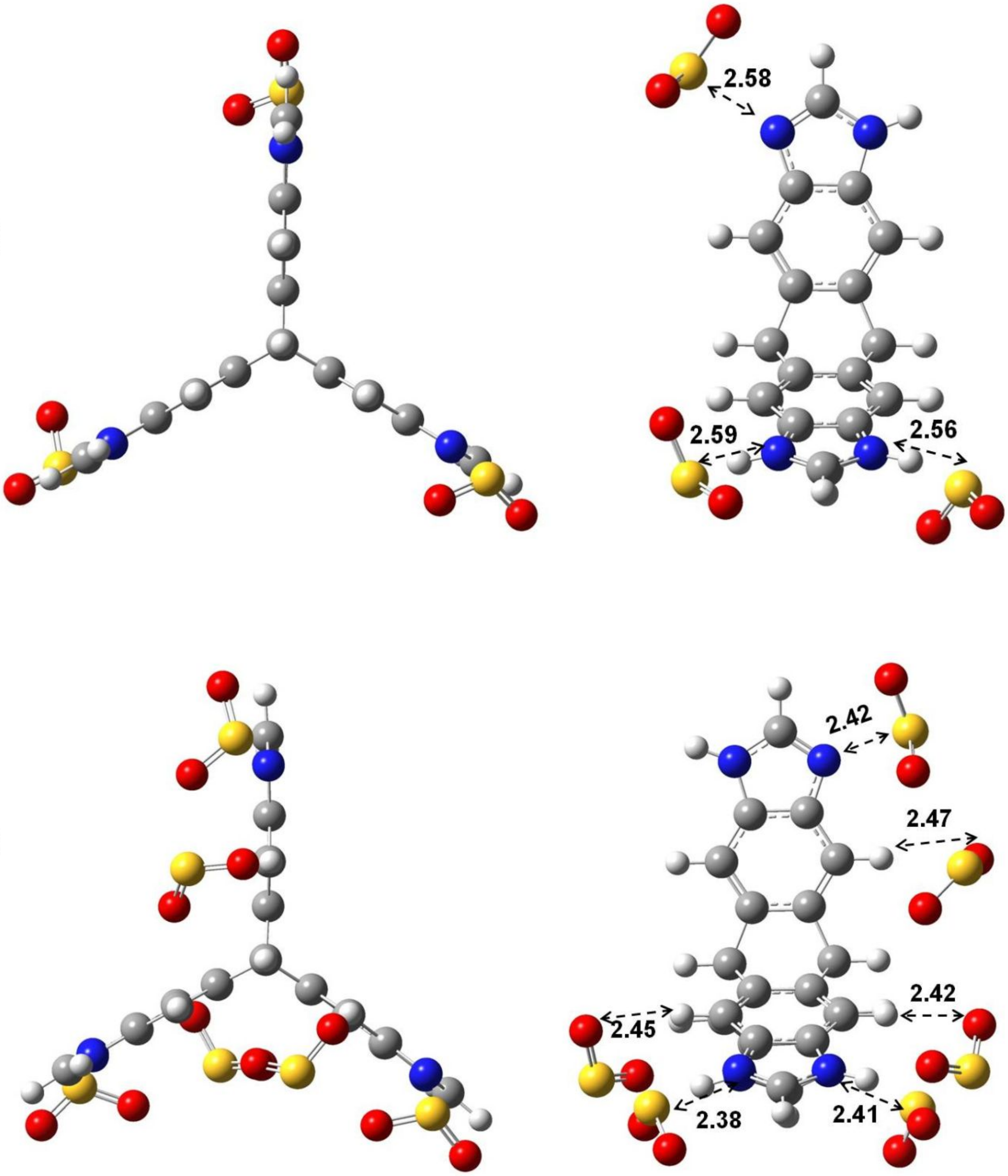

Figure S11. Partially optimized geometries of (a) BILP-3@3SO_2 and (b) BILP-3@6SO_2 calculated at LDA/6-311+G* level of theory. These were obtained by carrying out constrained optimization of BILP-3@3SO_2, where we froze the BILP-3 segment and only allowed optimization of binding sites of the SO_2 molecules. The bond lengths are in Å. Front and side views are given on the left and right panel, respectively. The blue, grey, white, yellow and red colors stand for N, C, H, S and O atoms.

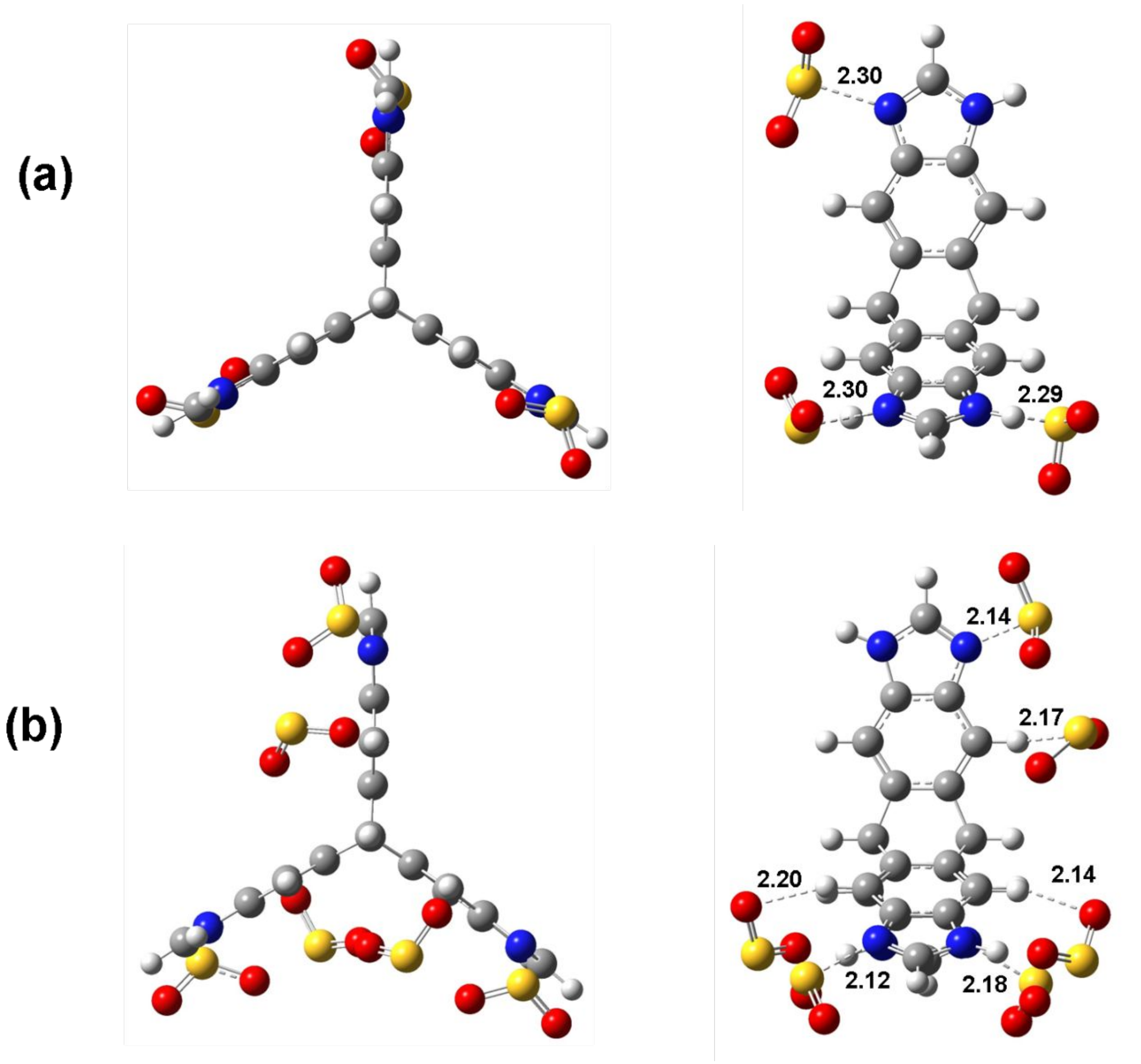

Selectivity Studies

Selectivity was calculated based on the initial slope calculations. The adsorption isotherms were fitted to dual-sight Langmuir Freundlich model (for SO_2, CO_2 and CH_4) or single site (for N_2). The fitted isotherms were used to calculate the selectivity.

Figure S12. Adsorption selectivity for (A) BILP-3, and (B) BILP-4 from initial slope calculations using pure component isotherms. Fitted isotherms from Figure 5 were used for initial slope calculations.

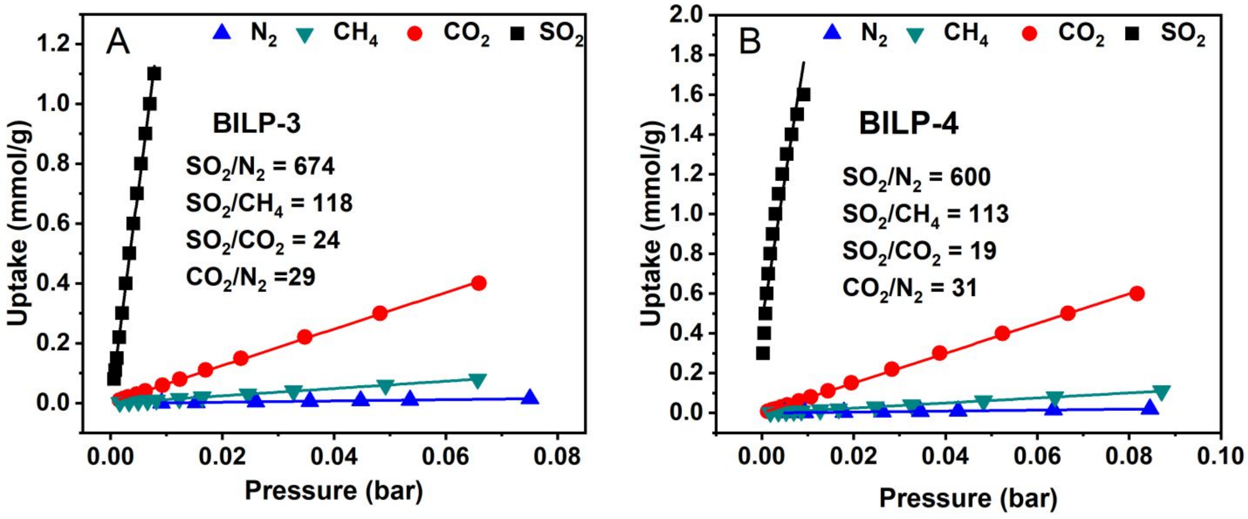

REFERENCES

1. Britt, D.; Tranchemontagne, D.; Yaghi, O. M., Metal-organic frameworks with high capacity and selectivity for harmful gases. *Proceedings of the National Academy of Sciences* **2008,** *105* (33), 11623-11627.
2. Fernandez, C. A.; Thallapally, P. K.; Motkuri, R. K.; Nune, S. K.; Sumrak, J. C.; Tian, J.; Liu, J., Gas-Induced Expansion and Contraction of a Fluorinated Metal−Organic Framework. *Crystal Growth & Design* **2010,** *10* (3), 1037-1039.
3. Thallapally, P. K.; Motkuri, R. K.; Fernandez, C. A.; McGrail, B. P.; Behrooz, G. S., Prussian Blue Analogues for CO2 and SO2 Capture and Separation Applications. *Inorganic Chemistry* **2010,** *49* (11), 4909-4915.
4. Yang, S.; Liu, L.; Sun, J.; Thomas, K. M.; Davies, A. J.; George, M. W.; Blake, A. J.; Hill, A. H.; Fitch, A. N.; Tang, C. C.; Schröder, M., Irreversible Network Transformation in a Dynamic Porous Host Catalyzed by Sulfur Dioxide. *Journal of the American Chemical Society* **2013,** *135* (13), 4954-4957.
5. Yang, S.; Sun, J.; Ramirez-Cuesta, A. J.; Callear, S. K.; DavidWilliam, I. F.; Anderson, D. P.; Newby, R.; Blake, A. J.; Parker, J. E.; Tang, C. C.; Schröder, M., Selectivity and direct

visualization of carbon dioxide and sulfur dioxide in a decorated porous host. *Nat Chem* **2012,** *4* (11), 887-894.
6. Valencia-Loza, S. d. J.; López-Olvera, A.; Martínez-Ahumada, E.; Martínez-Otero, D.; Ibarra, I. A.; Jancik, V.; Percástegui, E. G., SO2 Capture and Oxidation in a Pd6L8 Metal–Organic Cage. *ACS Applied Materials & Interfaces* **2021,** *13* (16), 18658-18665.
7. Martínez-Ahumada, E.; He, D.; Berryman, V.; López-Olvera, A.; Hernandez, M.; Jancik, V.; Martis, V.; Vera, M. A.; Lima, E.; Parker, D. J.; Cooper, A. I.; Ibarra, I. A.; Liu, M., SO2 Capture Using Porous Organic Cages. *Angewandte Chemie International Edition* **2021,** *60* (32), 17556-17563.
8. Savage, M.; Cheng, Y.; Easun, T. L.; Eyley, J. E.; Argent, S. P.; Warren, M. R.; Lewis, W.; Murray, C.; Tang, C. C.; Frogley, M. D.; Cinque, G.; Sun, J.; Rudić, S.; Murden, R. T.; Benham, M. J.; Fitch, A. N.; Blake, A. J.; Ramirez-Cuesta, A. J.; Yang, S.; Schröder, M., Selective Adsorption of Sulfur Dioxide in a Robust Metal–Organic Framework Material. *Advanced Materials* **2016,** *28* (39), 8705-8711.
9. Brandt, P.; Nuhnen, A.; Lange, M.; Möllmer, J.; Weingart, O.; Janiak, C., Metal–Organic Frameworks with Potential Application for SO2 Separation and Flue Gas Desulfurization. *ACS Applied Materials & Interfaces* **2019,** *11* (19), 17350-17358.
